# Preventive Effects of a Chinese Herbal Formula, Shengjiang Xiexin Decoction, on Irinotecan-Induced Delayed-Onset Diarrhea in Rats

**DOI:** 10.1155/2017/7350251

**Published:** 2017-01-12

**Authors:** Chao Deng, Bo Deng, Liqun Jia, Huangying Tan, Pan Zhang, Sida Liu, Yanan Zhang, Aiping Song, Lin Pan

**Affiliations:** ^1^School of Clinical Chinese Medicine, Beijing University of Chinese Medicine, Beijing 100029, China; ^2^Department of Oncology of Integrative Chinese and Western Medicine, China-Japan Friendship Hospital, Beijing 100029, China

## Abstract

Irinotecan is a well-known chemotherapy drug for the treatment of various cancers. However, delayed-onset diarrhea is a common adverse reaction, limiting the application of the drug. The study presented was designed to evaluate the preventive effects of Shengjiang Xiexin decoction (SXD) on irinotecan-induced diarrhea and to explore the possible mechanisms of this action. We established a diarrhea rat model. The condition of the rats was observed. The proliferation and apoptosis of intestinal cells were measured using immunohistochemical assays and a caspase-3 activity assay, respectively. The expression of Lgr5 and CD44 staining were used to observe intestinal stem cells (ISCs). In addition, the activity of *β*-glucuronidase in the rats' feces was measured. Our results showed that the number of proliferating intestinal cells in the SXD groups was obviously higher, while the activity of caspase-3 was lower. The expression of Lgr5 and the integrated option density (IOD) of CD44 stain were increased significantly by SXD. Additionally, SXD decreased the activity of *β*-glucuronidase after irinotecan administration. In conclusion, SXD exhibited preventive effects on irinotecan-induced diarrhea, and this action was associated with an inhibitory effect on intestinal apoptosis and *β*-glucuronidase and a promotive effect on intestinal cell proliferation due to increased maintenance of ISCs.

## 1. Background and Introduction

Irinotecan (CPT-11) is a camptothecin-derived cytotoxic anticancer drug. As a DNA topoisomerase I inhibitor, it inhibits the replication of tumor cells during the proliferation phase [1]. It is used clinically in the chemotherapy of colorectal cancer, breast cancer, and lung cancer [2–4]. Delayed-onset diarrhea is a common adverse reaction to irinotecan; occurs more than 24 hours after administration of irinotecan at all dose levels [5]. The incidence rate of grade 3 or 4 diarrhea is 16%–22% for chemotherapy using irinotecan alone and 11–14% for combination therapy including irinotecan, such as the FORFIRI regimen [5, 6].

Irinotecan is metabolized in the liver, where carboxylesterase (CESs) catalyzes irinotecan into SN-38, a metabolite with higher cytotoxicity than irinotecan itself [7]. SN-38 is inactivated to SN-38G in the liver by uridine diphosphate glucuronic acid transferases (UGTs) [8]. Then irinotecan and its metabolites are transported to the small intestine through biliary excretion. In the intestinal tract, SN-38G is hydrolyzed back to SN-38 through the action of *β*-glucuronidase produced by the intestinal bacteria [9]. Accumulation of SN-38 in the intestinal tract then causes intestinal mucosal injury, resulting in delayed-onset diarrhea.

Although the National Comprehensive Cancer Network (NCCN) has not yet released guidelines regarding chemotherapy-induced diarrhea, several recommendations have been published representing expert consensus on the prevention, management, and treatment of irinotecan-induced diarrhea based on evidences from preliminary clinical trials [10–12]. Octreotide and the opioid loperamide are generally recommended for irinotecan-induced diarrhea. However, in clinical practice, the failure rate of loperamide treatment is high [13], and the benefits of octreotide are controversial [14]. Many other medicines have potential efficacy for irinotecan-induced diarrhea, including budesonide [15], antibiotics [16], probiotics [17], glutamine (Gln) [18, 19], and active carbon [20, 21]. The majority of these therapies are still in the preclinical study phase, and results thus far have been inconsistent.

The pathogenesis of irinotecan-induced diarrhea is not yet thoroughly understood. It may involve multiple pathways, which could explain the limited efficacy of medicines with a single action. Traditional Chinese medicine (TCM) has the potential to overcome this problem because of its multitarget effects. Several TCM prescriptions have been shown to have novel efficacy as antidiarrheal medicine in animal models and clinical practice. Hangeshashin-to (TJ-14) has reduced the frequency of grade 3 and 4 irinotecan-induced diarrhea in patients [22]. Dai-kenchu-to (DKT) played a role in maintaining mucosal integrity in an irinotecan-induced diarrhea model by suppressing inflammatory cytokines (IL-1*β* and IFN-*γ*) and apoptosis in the intestinal mucosa [23]. PHY906 restored the intestinal epithelium by promoting the regeneration of intestinal stem cells (ISCs) [24]. These results suggest that Chinese medicinal formulae are a promising approach for the prevention of irinotecan-induced delayed-onset diarrhea.

Shengjiang Xiexin decoction (SXD) is a TCM prescription used in the treatment of gastrointestinal disorders. The classic text,* Treatise on Febrile Diseases*, recommends using the decoction for “intestinal tract with moisture, borborygmus, and diarrhea.” According to the theories of TCM, the efficacy of SXD is due to “dissipation of water-dampness, harmonizing stomach, and elimination of lump.”

In our lab, SXD previously exhibited protective effects on bowel function cells (goblet cells, paneth cells, and endocrine cells) in rats [25] and also upregulated T-lymphocytes (CD4+ and CD8+) and sIgA in intestinal mucosa [26]. However, there is lack of data clarifying in vivo the effects of SXD on *β*-glucuronidase, which plays a key role in the formation of SN-38 from its glucuronide conjugate in intestinal tract. In the present study, we investigated the effects of SXD on fecal *β*-glucuronidase activity before and after irinotecan administration in rats. We believe that this study is the first to use a caspase-3 activity assay to investigate the inhibitory effects of SXD on intestinal apoptosis. The goal of this research is to explore the potential mode of action of SXD on irinotecan-induced intestinal injury in rats.

## 2. Materials and Methods

### 2.1. Grouping of Animals

Thirty male Sprague-Dawley rats, weighing 180 ± 20 g, were purchased from Beijing Vital River Experimental Animal Technique Ltd. Co. (License no. SCXK [Jing] 2011-0011). Rats were housed in the SPF Animal Department of Clinical Institute of China-Japan Friendship Hospital (License no. SYXK [Jing] 2015–0017) and supplied with food and water ad libitum. The rats were caged individually in a temperature-controlled room with a 12 : 12 h dark-light cycle. Using a random number table, the rats were divided into 5 groups of 6 rats each: a blank control group, a model control group, and low-dose, middle-dose, and high-dose SXD-treated groups.

### 2.2. Establishing Delayed-Onset Irinotecan-Induced Diarrhea Model

**Figure 1:**
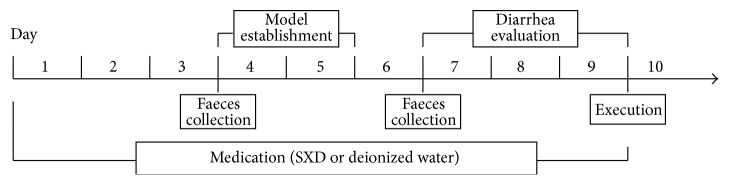
Diagrammatic sketch of experimental process.

Using the animal model described by Trifan et al. [27], each rat in the model control group and SXD-treated groups was injected with irinotecan (no. JH26A; Pfizer, USA; 150 mg/kg/day) in the tail vein for two consecutive days (day 4 and day 5; Figure 1). The rats in the blank control group were injected with isovolumetric normal saline (no. 5D83F2; Otsuka, China) in the same way.

### 2.3. Preparation of Shengjiang Xiexin Decoction (SXD)

SXD comprises eight Chinese herbs, as shown in Table 1. All herbs were supplied by the TCM Pharmacy of China-Japan Friendship Hospital. Seven times the normal dosage for adult humans was defined as middle dosage of SXD for rats. Thus, rats in the low-dose, middle-dose, and high-dose SXD groups were given 5 g/kg, 10 g/kg, and 15 g/kg SXD, respectively. The SXD was condensed to the corresponding concentrations of 1 g/mL, 2 g/mL, and 3 g/mL in a water bath. The concentration of SXD represents the dry weight of the raw herbs used to produce one milliliter of decoction.

### 2.4. Methods of Medication

Medication started 3 d before irinotecan administration and continued to the fourth day after the last irinotecan dose was administered, a total of 9 d (Figure 1). The SXD was administered intragastrically to the rats in the three SXD-treated groups one time per day. Rats in the blank control and model control groups received isovolumetric deionized water in the same way.

### 2.5. Observation of the Condition of Rats

The general condition of each rat was observed at 8:00 AM every day, including body weight (g) and food intake (g). Beginning 24 h after the last irinotecan dose (day 7 to day 9), the severity of delayed-onset diarrhea was recorded at 8:00 AM and 8:00 PM each day (Figure 1). Diarrhea was evaluated using the scale described by Kurita et al. [28], as shown in Table 2.

### 2.6. Histomorphology of Intestinal Mucosa

All rats were sacrificed on the fifth day after the last dose of irinotecan (day 10). Jejunum tissues were extracted, immediately fixed with formalin, and embedded in paraffin. The histological structure of the jejunum was observed under a microscope with haematoxylin and eosin (HE) staining, including the architecture of the intestinal epithelium, the length of villi, and the number of intestinal crypts.

### 2.7. Intestinal Cell Apoptosis Assay

Caspase-3 is an important enzyme in the process of apoptosis. Apoptosis of the intestinal cells was detected using a caspase-3 activity assay kit (Beyotime, China). In this assay, caspase-3 catalyzes the substrate acetyl-Asp-Glu-Val-Asp* p*-nitroanilide (Ac-DEVD-*p*NA) to produce* p*-nitroaniline (*p*NA), and the amount of* p*NA present is measured spectrophotometrically to determine the activity of caspase-3. Briefly, intestinal tissues were homogenized with lysis buffer (0.10 mg/*μ*L) and then centrifuged at 20000*g* for 15 min at 4°C. Then 10 *μ*L of the supernatant was incubated with 80 *μ*L buffer, 10 *μ*L Ac-DEVD-*p*NA (2 mM) for 60 min at 37°C. Then the absorbance of the reaction product,* p*NA, was measured at 405 nm in a spectrophotometer (Multiskan Spectrum MK3, Thermo Fisher Scientific, USA). The amount of* p*NA in the sample was calculated according to a standard curve of* p*NA. The amount of caspase-3 that catalyzed the substrate to produce 1 nmol* p*NA per hour at 37°C was defined as one unit.

### 2.8. Intestinal Cell Proliferation Assays

Immunohistochemical assays for proliferating cell nuclear antigen (PCNA) and the homing cell adhesion molecule CD44 were performed to quantify the proliferation of intestinal epithelium cells and ISCs. The primary antibodies used were as follows: anti-PCNA (Santa Cruz, USA) diluted to 1 : 6400 and anti-CD44 (Santa Cruz, USA) diluted to 1 : 200. Paraffin blocks containing embedded jejunum tissues (see Section 2.6) were sliced into sections. The sections were deparaffinized, hydrated, and immersed first in 3% H_2_O_2_ solution for 10 min and then in phosphate buffered saline (PBS) for 5 min. Then the tissue sections were incubated with the primary antibodies for 60 min. After that, the tissue sections were immersed for 5 min in PBS three times. Then the second antibody, EnVision/HRP (DAKO, Denmark), was added to the tissue sections and allowed to incubate for 20 min. Then the tissue sections were again immersed in PBS for 5 min three times, color development with DAB-H_2_O_2_ for 1–5 min. The tissue sections were then processed by washing, counterstaining, dehydration, and transparency before being mounted. Three views were randomly chosen per tissue section for photography with a digital imaging system. The integrated option density (IOD) of PCNA and CD44 staining per view was measured using Image-Pro Plus (IPP) software (Media Cybernetics, USA). The expression of PCNA was also quantified by counting the number of positively stained cells per view.

As another marker of ISCs in crypts, mRNA expression of Lgr5 was detected by real-time PCR. Briefly, total RNA of intestinal tissue was extracted by Trizol (Invitrogen, USA). cDNA was synthesized using the 1st Strand cDNA Synthesis Kit (Takara, Japan) according to the manufacturer's instructions. Real-time PCR was performed in a 10 *μ*L reaction system, including 3.6 *μ*L RNase-free ddH2O, 5.0 *μ*L 2 × UltraSYBR Mixture, 0.2 *μ*L each forward and reverse primers (0.2 *μ*M), and 1.0 *μ*L cDNA (1 ng/*μ*L). The primer sequences were as follows: Lgr5, 5′-TGC CAT TAT TCA CCC CAA C-3′ (forward), 5′-CAC AGC ACT GGT AAG CGT ATG-3′ (reverse); rGAPDH, 5′-CTC AAC TAC ATG GTC TAC ATG TTC CA-3′ (forward), 5′-CTT CCC ATT CTC AGC CTT GAC T-3′ (reverse). Thermal cycles were 10 min at 95°C initially, followed by 40 cycles of 15 s at 95°C and 1 min at 60°C in an ABI 7900HT real-time quantitative PCR system (Applied Biosystems, USA). The threshold cycle (CT) was recorded to express the value of each sample (triplicate for mean value). Relative quantification of Lgr5 mRNA was normalized using an internal control rGAPDH gene, according to the comparative CT method [29].

### 2.9. Fecal *β*-Glucuronidase Assay

Activity of *β*-glucuronidase in the rats' feces was measured using the method described by Shiau and Chang [30]. Briefly, fresh fecal pellets were collected in the morning before and after irinotecan administration (day 4 and day 7), then weighed, and mixed with PBS (0.01 M, pH 7.5) at a wt/wt ratio of 1 : 100. After softening for 20 min, the fecal pellets were homogenized. Then 0.05 mL 4-nitrophenyl *β*-D-glucuronide (0.01 M, Sigma, USA) was added to 1 mL diluted fecal homogenate to produce the following reaction:

4-nitrophenyl *β*-D-glucuronide + *β*-glucuronidase → 4-nitrophenol + *β*-glucuronide

All reaction solutions were incubated at 37°C for 60 min and then centrifuged at 3000*g* for 10 min. The absorbance of each supernatant solution was measured at 405 nm in a spectrophotometer (Multiskan Spectrum GO, Thermo Fisher Scientific, USA). The concentration of 4-nitrophenol in each sample was calculated by reference to a standard curve. One unit of fecal *β*-glucuronidase was defined as the amount of enzyme that will cleave 1 nmol 4-nitrophenol per hour at 37°C under saturated substrate concentrations.

### 2.10. Statistical Analysis

All statistical analyses were carried out using the Statistical Package of Social Science (SPSS) software version 19.0 (IBM, USA). Differences were considered significant at *p* < 0.05. Intergroup comparisons were made using ANOVA analysis with the Dunnett method. Ranked data and data with nonnormal distribution or unequal variance were analyzed using the Kruskal-Wallis H rank-sum test.

## 3. Results

### 3.1. Condition of the Rats

**Figure 2:**
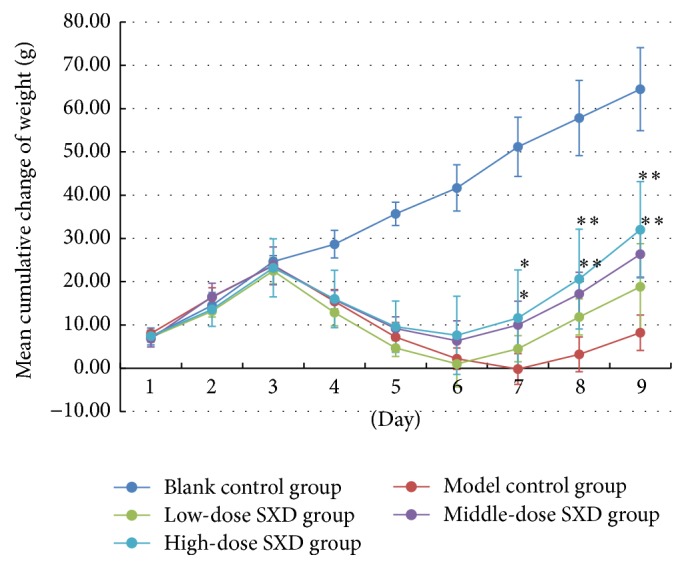
Effects of SXD on the weight of rats after irinotecan administration. Notes: the data were expressed as mean ± SD. ^*∗*^*p* < 0.05, ^*∗∗*^*p* < 0.01, compared with the model group.

Two rats died during the injection of irinotecan (day 4 and day 5), one in the model control group and one in the high-dose SXD group. Data on the dead rats were not used in the statistical analysis. SXD ameliorated the loss of weight associated with the injection of irinotecan. As shown in Figure 2, the rats in the model control group and the SXD groups lost weight beginning on day 4. On day 7, day 8, and day 9, the cumulative-added weight was significantly higher in the high-dose and middle-dose SXD groups than it was in the model control group (*p* < 0.05, day 7; *p* < 0.01, day 8 and day 9). However, differences between the groups receiving different doses of SXD were not significant, until day 9, when the rats in the high-dose SXD group had significantly higher cumulative-added weight than the rats in the low-dose SXD group (*p* < 0.05).

**Figure 3:**
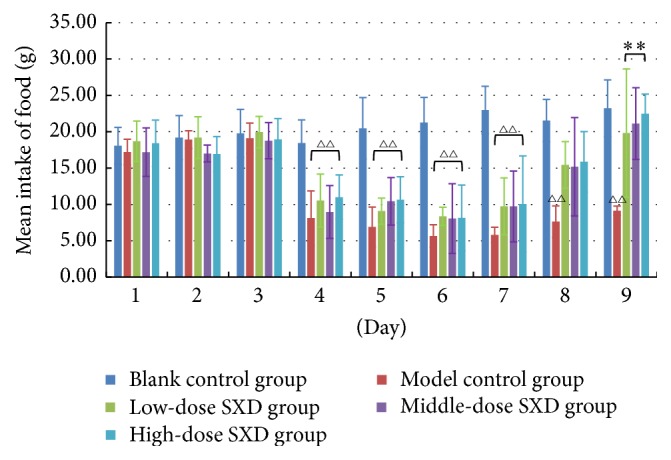
Effects of SXD on the food intake of rats after irinotecan administration. Notes: the data were expressed as mean ± SD. ^△△^*p* < 0.01, compared with blank control group; ^*∗∗*^*p* < 0.01, compared with the model group.

Irinotecan administration was associated with decreased food intake, and treatment with SXD promoted the recovery of intake. As shown in Figure 3, the model control and SXD groups had significantly lower intake of food since day 4 (*p* < 0.01) compared with the blank control group. On day 9, intake of food in the SXD groups was significantly higher than it was in the model control group (*p* < 0.01), but differences between the groups receiving different doses of SXD were not significant.

**Figure 4:**
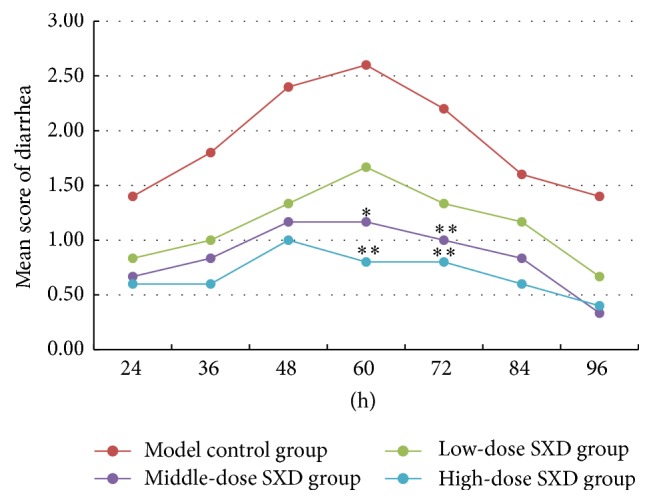
Effects of SXD on the grade and score of diarrhea after irinotecan administration. Notes: the data were expressed as mean. Distribution of diarrhea grade was analyzed by Kruskal-Wallis H rank-sum test. ^*∗*^*p* < 0.05, ^*∗∗*^*p* < 0.01, compared with the model group.

The mean score of diarrhea began to rise in the model control and SXD groups after 24 hours from the last irinotecan injection (Figure 4). After 60 and 72 hours, differences in distribution of diarrhea grade were significant between the middle-dose SXD group and the model control group (*p* < 0.05, *p* < 0.01) and between the high-dose SXD group and the model control group (*p* < 0.01). The SXD groups had a lower mean score of diarrhea than the model control group. These results are evidence that SXD decreases the grade and score of irinotecan-induced diarrhea.

### 3.2. Histomorphology of Intestinal Mucosa

**Figure 5:**
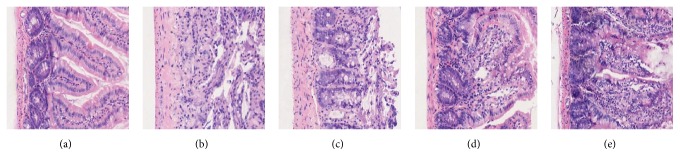
Effects of SXD on the intestinal mucosa of rats after irinotecan administration (HE ×100). (a) Blank control group: the architecture of the intestinal epithelium has integrity and intestinal crypts and villi can be seen clearly. (b) Model control group: the architecture of the epithelium is destroyed, epithelial cell arrangement is disordered, and intestinal crypts and villi are reduced. (c) Low-dose SXD group, (d) middle-dose SXD group, and (e) high-dose SXD group: intestinal crypts are maintained; the general architecture of the epithelium and the outline of the intestinal villi can be seen indistinctly.

SXD exhibited a protective effect on the intestinal mucosa by decreasing the destruction of histological structure caused by irinotecan. As shown in Figure 5, the architecture of the intestinal epithelium in the SXD group rats had more integrity than that of the model control group. The intestinal crypts of the SXD group rats were also better maintained, which is important for the regeneration of intestinal cells.

### 3.3. Apoptosis of Intestinal Cells

SXD inhibited the apoptosis of intestinal cells, which was revealed by the activity of intestinal caspase-3. As shown in Table 3, the enzymatic activity of intestinal caspase-3 was significantly decreased in the SXD groups, though no differences were evident between the dose groups.

### 3.4. Proliferation of Intestinal Cells

**Figure 6:**
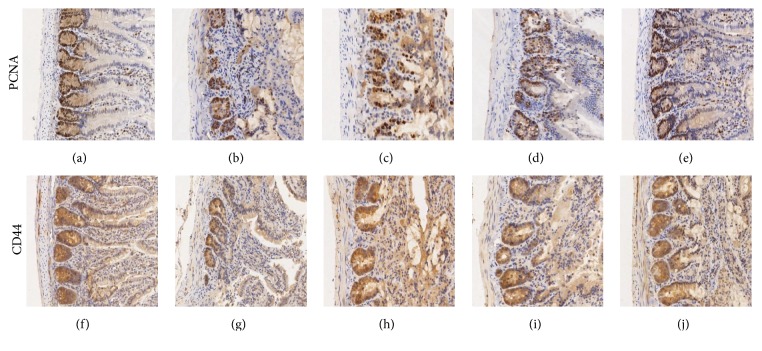
Effects of SXD on intestinal proliferating cells and ISCs of rats after irinotecan administration (×100). ((a) and (f)) Blank control group; ((b) and (g)) model control group; ((c) and (h)) low-dose SXD group; ((d) and (i)) middle-dose SXD group; ((e) and (j)) high-dose SXD group.

SXD promoted the regeneration of intestinal epithelium by increasing the proliferation of intestinal cells, particularly ISCs. We used PCNA to label the nuclei of proliferating cells and CD44 to label the cytomembranes of ISCs. As shown in Table 4 and Figure 6, irinotecan suppressed the proliferation of intestinal epithelium cells and ISCs. The number of positive cells and the IOD of PCNA were higher in the SXD groups than in the model control group and IOD of CD44 staining as well. In particular, the high-dose of SXD markedly increased the expression of PCNA and CD44. The high-dose SXD group had significantly higher expression of PCNA than the low-dose SXD group, but there were no obvious differences in the expression of CD44 in ISCs among SXD groups.

**Figure 7:**
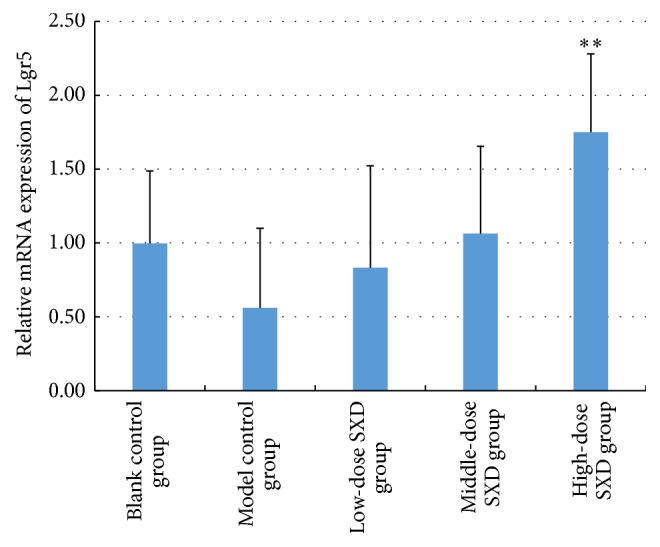
Effects of SXD on the expression of Lgr5 mRNA in the intestinal tissue. Notes: the data were expressed as mean ± SD. ^*∗∗*^*p* < 0.01, compared with the model control group.

As shown in Figure 7, irinotecan reduced the expression of Lgr5 mRNA, but this effect was significantly lessened in the high-dose SXD group (*p* < 0.01, compared with the model control group). Low-dose and middle-dose SXD did not exhibit an obvious effect on Lgr5 mRNA.

### 3.5. Activity of Fecal *β*-Glucuronidase

As shown in Table 3, low- and middle-dose SXD treatment had no obvious effect on fecal *β*-glucuronidase prior to irinotecan administration, while high-dose SXD did have an effect. After irinotecan administration, fecal *β*-glucuronidase activity in the SXD groups was lower than it was in the model control group prominently. There were no significant differences between the SXD groups, suggesting that the inhibitory effect of SXD on fecal *β*-glucuronidase did not perform in a dose-dependent manner.

## 4. Discussion

TCM has exhibited efficacy in the prophylaxis and treatment of chemotherapy-induced side effects [31]. As an adjuvant method, it can improve the tolerance to chemotherapy and thus improve cancer patients' quality of life. SXD is a classical TCM prescription for the treatment of gastrointestinal diseases. In an early clinical observation, SXD was shown to have a preventive effect on irinotecan-induced delayed-onset diarrhea. However, the mechanism of action of SXD is not well understood. In the present study, the effects of SXD on intestinal cells and intestinal microflora were investigated in a rat model.

In the research presented here, SXD decreased the grade of irinotecan-induced diarrhea in rats and also mitigated the reduction in body weight and food intake associated with irinotecan administration. The results show efficacy in alleviating enterotoxigenesis caused by irinotecan, an effect that did not appear to be dose-dependent in the dose range studied.

The occurrence of diarrhea has a close relationship with injury to intestinal cells, and intestinal homeostasis is normally maintained by a dynamic balance between the proliferation and apoptosis of intestinal cells [32]. The enterotoxigenesis of irinotecan involves increasing the rate of intestinal cell apoptosis, thus disrupting the intestinal cell homeostasis. Previous research has shown that SXD protects the integrity of intestinal epithelial cells and bowel function cells in a rat model of diarrhea [25]. In the research described here, proliferating cells in the intestinal structure were labeled by immunohistochemical staining. The number of proliferating cells in the SXD-treated groups was obviously higher than the number in the model control group. The activity of caspase-3, a key enzyme in the process of apoptosis, was assayed to quantify intestinal cell apoptosis [33]. We found that the activity of caspase-3 in the SXD-treated groups was lower than it was in the model control group, though differences between the dosages tested were insignificant.

Intestinal epithelial cells are maintained by ISCs located in the crypts, and the process of renewal is rapid [34, 35]. Injury to the ISCs affects the regeneration of the intestinal epithelium and destroys the homeostasis of the intestinal tract [36, 37]. In our previous work, we found that bowel function cells, which are differentiated from ISCs, decreased in number after rats were injected with irinotecan, and that SXD mitigated this reduction [25]. So we further hypothesized that SXD has protective effects on ISCs. Several ISC markers have been proposed, but the validity of most of these markers has not yet been confirmed [38]. Lgr5 has been shown to be expressed exclusively in cycling columnar cells at the crypts [39], and for this reason it is regarded as a marker for ISCs. In this study, we observed upregulation in the expression of Lgr5 mRNA in the high-dose SXD group compared with the model control group. Further study with more samples is needed to confirm these results. Intestinal crypts containing ISCs also have a high expression of the cell surface antigen CD44 [40], so CD44 has also been used for identification of putative ISCs [41]. We observed higher expression of Lgr5 and CD44 in SXD-treated groups, which suggests that SXD has a protective effect on ISCs. Further evidence is needed to verify this hypothesis.

Along with intestinal cells, intestinal microbiota play an important role in maintaining intestinal homeostasis. Microbiota can influence the efficacy and toxicity of drugs through regulating the levels of metabolic enzymes and transporters [42, 43]. The inactivated metabolite of irinotecan, SN-38G, is reactivated to SN-38 by bacterial *β*-glucuronidase in the intestinal tract [9]. Irinotecan can alter the structure of the intestinal microbial community, increasing the activity of *β*-glucuronidase and thus increasing the toxicity of irinotecan [44]. For this reason, antibiotics have been considered as a potential way to alleviate irinotecan-induced diarrhea [16]. However, antibiotics inevitably kill bacteria that are beneficial for human health, and this can cause diarrhea or dysbacteriosis [45]. Moreover, a large portion of the SN-38 in the gut may be absorbed by intestinal microflora and dietary fibers, due to the high hydrophobicity of SN-38 [46]. Therefore, more evidence is required to determine if reducing intestinal bacteria is beneficial for the treatment of irinotecan-induced diarrhea. A targeted approach that inhibits *β*-glucuronidase without killing beneficial bacteria would be ideal [47]. Investigations into *β*-glucuronidase inhibitors are in progress, some involving a synthetic compound in the experimental stage [47, 48] and others involving known drugs used to treat depression, such as nialamide, phenelzine, and amoxapine [49, 50]. In addition, baicalin, a compound that occurs naturally in the plant* Scutellaria baicalensis*, has shown an inhibiting effect on *β*-glucuronidase in vitro studies [51]. In the present study, SXD, which contains baicalin, also exhibited this effect. At all of the dosages tested, SXD decreased the activity of *β*-glucuronidase in the intestinal tract of rats injected with irinotecan. And, except for the highest-dose tested, SXD had no obvious inhibiting effect on *β*-glucuronidase in rats before irinotecan was administered. These results suggest that SXD has a balancing effect on changes in *β*-glucuronidase activity resulting from irinotecan administration.

The accumulation of SN-38 is believed to cause injury to the intestine epithelia directly. Therefore, along with biliation and reactivation by *β*-glucuronidase, the absorption of SN-38 by intestinal tissues is also an important factor in irinotecan-induced diarrhea. In addition to the *β*-glucuronidase-inhibiting effect mentioned above, baicalin could also suppress the organic anion transporting polypeptide (OATP) 2B1, one of intestinal transporters, which contributes to the uptake of SN-38 in the intestinal tract [52].

Another constituent of SXD, berberine, has been used in the treatment of diarrhea in China for a long time. The efficacy of berberine in the treatment of diarrhea has been validated in clinical studies [53]. A review has summarized the potential mechanisms by which berberine alleviates disruption of the intestinal epithelium and improves the symptoms of diarrhea, including a bactericidal effect on* E. coli*, inhibition of intestinal motility, and reduction of intestinal secretion and epithelial permeability [54].

The results of this study show that SXD has a protective effect on irinotecan-induced diarrhea and shed light on the possible mechanisms for this effect. The results did not successfully illuminate a dose-effect relationship for SXD, and the scope of this study only targeted the intestinal tract, ignoring the liver, the main site of irinotecan metabolism. More studies are needed to further explore the effects of SXD on the metabolic enzymes that act on irinotecan in the liver, such as CESs and UGTs.

## 5. Conclusion

In summary, SXD inhibited the apoptosis of intestinal cells and contributed to the repair of intestinal injury through promoting the proliferation of intestinal cells. In addition, SXD inhibited the activity of *β*-glucuronidase, which is a key factor in the accumulation of the toxic irinotecan metabolite SN-38. These data may help to explain the effect of SXD in ameliorating delayed-onset diarrhea induced by irinotecan.

## Figures and Tables

**Table 1:** Constituents of SXD.

Chinese name	Botanical Latin name	Weight(g)
Shengjiang	*Rhizoma Zingiberis Recens*	12
Ganjiang	*Rhizoma Zingiberis*	3
Huangqin	*Radix Scutellariae*	9
Huanglian	*Rhizoma Coptidis*	3
Banxia	*Rhizoma Pinelliae*	9
Dangshen	*Radix Codonopsis*	9
Dazao	*Fructus Jujubae*	12
Gancao	*Radix Glycyrrhizae*	9

**Table 2:** Evaluation scale for irinotecan-induced delayed-onset diarrhea in rats.

Extent of diarrhea	Manifestations	Score
Normal	Normal stool or absent	0
Slight	Slightly wet and soft stool	1
Moderate	Wet and unformed stool with moderate perianal staining of the coat	2
Severe	Watery stool with severe perianal staining of the coat	3

**Table 3:** Comparison of the activity of intestinal caspase-3 and fecal *β*-glucuronidase in different groups.

Group	*N*	Activity of intestinal caspase-3 (U)	Activity of fecal *β*-glucuronidase (U)	
			Before irinotecan administration	After irinotecan administration
Blank control group	6	0.45 ± 0.26^*∗∗*^	5.76 ± 1.24	8.00 ± 2.31^*∗∗*^
Model control group	5	1.00 ± 0.17^△△^	5.92 ± 1.47	16.09 ± 1.40^△△^
Low-dose SXD group	6	0.66 ± 0.10^△*∗∗*^	5.80 ± 1.55	12.56 ± 1.81^△△*∗∗*^
Middle-dose SXD group	6	0.48 ± 0.11^*∗∗*^	4.98 ± 1.24	13.55 ± 1.17^△△*∗*^
High-dose SXD group	5	0.68 ± 0.19^△*∗∗*^	3.88 ± 1.78^△*∗*^	11.62 ± 1.78^△△*∗∗*^

Notes: one unit of enzyme activity is the amount of enzyme that will cleave 1 nmol of the colorimetric substrate per hour at 37°C under saturated substrate concentrations. The data were expressed as mean ± SD. ^△^*p* < 0.05, ^△△^*p* < 0.01, compared with blank control group; ^*∗*^*p* < 0.05, ^*∗∗*^*p* < 0.01, compared with the model group.

**Table 4:** Comparison of the expression of PCNA and CD44 per view in intestinal sections in different groups.

Group	*N*	PCNA		CD44
		Number of positive cells	IOD	IOD
Blank control group	6	650.00 ± 88.91^*∗∗*^	3633.73 ± 814.00^*∗∗*^	697.02 ± 145.83^*∗∗*^
Model control group	5	312.40 ± 43.23^△△^	1026.26 ± 339.00^△△^	360.99 ± 156.87^△△^
Low-dose SXD group	6	380.67 ± 97.93^△△^	1586.32 ± 220.94^△△^	507.53 ± 87.29^△^
Middle-dose SXD group	6	409.33 ± 45.13^△△^	2006.49 ± 250.84	511.35 ± 83.18^△*∗*^
High-dose SXD group	5	531.20 ± 101.64^△*∗∗*^	2494.93 ± 615.00^*∗*^	613.04 ± 102.42^*∗∗*^

Notes: the data were expressed as mean ± SD. ^△^*p* < 0.05, ^△△^*p* < 0.01, compared with blank control group; ^*∗*^*p* < 0.05, ^*∗∗*^*p* < 0.01, compared with the model group.

## References

[1] Liu Y.-Q., Li W.-Q., Morris-Natschke S. L. (2015). Perspectives on biologically active camptothecin derivatives. *Medicinal Research Reviews*.

[2] Van Cutsem E., Lenz H.-J., Köhne C.-H. (2015). Fluorouracil, leucovorin, and irinotecan plus cetuximab treatment and RAS mutations in colorectal cancer. *Journal of Clinical Oncology*.

[3] Hayashi H., Tsurutani J., Satoh T. (2013). Phase II study of bi-weekly irinotecan for patients with previously treated HER2-negative metastatic breast cancer: KMBOG0610B. *Breast Cancer*.

[4] Satouchi M., Kotani Y., Shibata T. (2014). Phase III study comparing amrubicin plus cisplatin with irinotecan plus cisplatin in the treatment of extensive-disease small-cell lung cancer: JCOG 0509. *Journal of Clinical Oncology*.

[5] Stein A., Voigt W., Jordan K. (2010). Chemotherapy-induced diarrhea: pathophysiology, frequency and guideline-based management. *Therapeutic Advances in Medical Oncology*.

[6] Muehlbauer P. M., Thorpe D., Davis A., Drabot R., Rawlings B. L., Kiker E. (2009). Putting evidence into practice: evidence-based interventions to prevent, manage, and treat chemotherapy- and radiotherapy-induced diarrhea. *Clinical Journal of Oncology Nursing*.

[7] Hicks L. D., Hyatt J. L., Stoddard S. (2009). Improved, selective, human intestinal carboxylesterase inhibitors designed to modulate 7-ethyl-10-[4-(1-piperidino)-1-piperidino]carbonyloxycamptothecin (irinotecan; CPT-11) toxicity. *Journal of Medicinal Chemistry*.

[8] Rouits E., Charasson V., Pétain A. (2008). Pharmacokinetic and pharmacogenetic determinants of the activity and toxicity of irinotecan in metastatic colorectal cancer patients. *British Journal of Cancer*.

[9] Yamamoto M., Kurita A., Asahara T. (2008). Metabolism of irinotecan and its active metabolite SN-38 by intestinal microflora in rats. *Oncology Reports*.

[10] Sharma R., Tobin P., Clarke S. J. (2005). Management of chemotherapy-induced nausea, vomiting, oral mucositis, and diarrhoea. *Lancet Oncology*.

[11] Maroun J. A., Anthony L. B., Blais N. (2007). Prevention and management of chemotherapy-induced diarrhea in patients with colorectal cancer: a consensus statement by the Canadian Working Group on Chemotherapy-Induced Diarrhea. *Current Oncology*.

[12] Benson A. B., Ajani J. A., Catalano R. B. (2004). Recommended guidelines for the treatment of cancer treatment-induced diarrhea. *Journal of Clinical Oncology*.

[13] Barbounis V., Koumakis G., Vassilomanolakis M., Demiri M., Efremidis A. P. (2001). Control of irinotecan-induced diarrhea by octreotide after loperamide failure. *Supportive Care in Cancer*.

[14] Hoff P. M., Saragiotto D. F., Barrios C. H. (2014). Randomized phase III trial exploring the use of long-acting release octreotide in the prevention of chemotherapy-induced diarrhea in patients with colorectal cancer: the LARCID trial. *Journal of Clinical Oncology*.

[15] Karthaus M., Ballo H., Abenhardt W. (2005). Prospective, double-blind, placebo-controlled, multicenter, randomized phase III study with orally administered budesonide for prevention of irinotecan (CPT-11)-induced diarrhea in patients with advanced colorectal cancer. *Oncology*.

[16] Takasuna K., Hagiwara T., Watanabe K. (2006). Optimal antidiarrhea treatment for antitumor agent irinotecan hydrochloride (CPT-11)-induced delayed diarrhea. *Cancer Chemotherapy and Pharmacology*.

[17] Mego M., Chovanec J., Vochyanova-Andrezalova I. (2015). Prevention of irinotecan induced diarrhea by probiotics: a randomized double blind, placebo controlled pilot study. *Complementary Therapies in Medicine*.

[18] Xue H., Sawyer M. B., Field C. J., Dieleman L. A., Murray D., Baracos V. E. (2008). Bolus oral glutamine protects rats against CPT-11-induced diarrhea and differentially activates cytoprotective mechanisms in host intestine but not tumor. *Journal of Nutrition*.

[19] Anami S., Saegusa K., Nishikata M. (2009). Effect of glutamine or alkaline ionized water on late diarrhea induced by irinotecan hydrochloride in Gunn rats. *Asian Journal of Pharmaceutical Sciences*.

[20] Sergio G.-C., Félix G.-M., Luis J.-V. (2008). Activated charcoal to prevent irinotecan-induced diarrhea in children. *Pediatric Blood & Cancer*.

[21] Michael M., Brittain M., Nagai J. (2004). Phase II study of activated charcoal to prevent irinotecan-induced diarrhea. *Journal of Clinical Oncology*.

[22] Mori K., Kondo T., Kamiyama Y., Kano Y., Tominaga K. (2003). Preventive effect of Kampo medicine (Hangeshashin-to) against irinotecan-induced diarrhea in advanced non-small-cell lung cancer. *Cancer Chemotherapy and Pharmacology*.

[23] Chikakiyo M., Shimada M., Nakao T. (2012). Kampo medicine “Dai-kenchu-to” prevents CPT-11-induced small-intestinal injury in rats. *Surgery Today*.

[24] Lam W., Bussom S., Guan F. (2010). Chemotherapy: the four-herb Chinese medicine PHY906 reduces chemotherapy-induced gastrointestinal toxicity. *Science Translational Medicine*.

[25] Wang J., Jia L. Q., Tan H. Y., Pan L., Yu L. L., Deng B. (2015). Effect of Shengjiang Xiexin decoction on the repair of damaged rat intestinal mucosa after irinotecan chemotherapy. *Zhongguo Zhong Xi Yi Jie He Za Zhi*.

[26] Deng H. Y., Jia L. Q., Pan L., Yu L. L. (2007). Effect of ShengJiangXieXinTang on the intestinal mucosal immune barrier of rats receiving irinotecan. *Chinese Journal of Immunology*.

[27] Trifan O. C., Durham W. F., Salazar V. S. (2002). Cyclooxygenase-2 inhibition with celecoxib enhances antitumor efficacy and reduces diarrhea side effect of CPT-11. *Cancer Research*.

[28] Kurita A., Kado S., Kaneda N., Onoue M., Hashimoto S., Yokokura T. (2000). Modified irinotecan hydrochloride (CPT-11) administration schedule improves induction of delayed-onset diarrhea in rats. *Cancer Chemotherapy and Pharmacology*.

[29] Schmittgen T. D., Livak K. J. (2008). Analyzing real-time PCR data by the comparative *C*_T_ method. *Nature Protocols*.

[30] Shiau S. Y., Chang G. W. (1983). Effects of dietary fiber on fecal mucinase and *β*-glucuronidase activity in rats. *The Journal of Nutrition*.

[31] Ohnishi S., Takeda H. (2015). Herbal medicines for the treatment of cancer chemotherapy-induced side effects. *Frontiers in Pharmacology*.

[32] Williams J. M., Duckworth C. A., Watson A. J. M. (2013). A mouse model of pathological small intestinal epithelial cell apoptosis and shedding induced by systemic administration of lipopolysaccharide. *Disease Models & Mechanisms*.

[33] Mazumder S., Plesea D., Almasan A. (2007). Caspase-3 activation is a critical determinant of genotoxic stress-induced apoptosis. *Methods in Molecular Biology*.

[34] Van Der Flier L. G., Clevers H. (2009). Stem cells, self-renewal, and differentiation in the intestinal epithelium. *Annual Review of Physiology*.

[35] Chia L. A., Kuo C. J. (2010). Chapter 7-the intestinal stem cell. *Progress in Molecular Biology and Translational Science*.

[36] Sipos F., Muzes G. (2015). Injury-associated reacquiring of intestinal stem cell function. *World Journal of Gastroenterology*.

[37] Yeung T. M., Chia L. A., Kosinski C. M., Kuo C. J. (2011). Regulation of self-renewal and differentiation by the intestinal stem cell niche. *Cellular and Molecular Life Sciences*.

[38] Montgomery R. K., Breault D. T. (2008). Small intestinal stem cell markers. *Journal of Anatomy*.

[39] Barker N., Van Es J. H., Kuipers J. (2007). Identification of stem cells in small intestine and colon by marker gene Lgr5. *Nature*.

[40] Chang P.-Y., Jin X., Jiang Y.-Y., Wang L.-X., Liu Y.-J., Wang J. (2016). Mensenchymal stem cells can delay radiation-induced crypt death: impact on intestinal CD44^+^ fragments. *Cell and Tissue Research*.

[41] Wang F., Scoville D., He X. C. (2013). Isolation and characterization of intestinal stem cells based on surface marker combinations and colony-formation assay. *Gastroenterology*.

[42] Swanson H. I. (2015). Drug metabolism by the host and gut microbiota: a partnership or rivalry?. *Drug Metabolism and Disposition*.

[43] Kang M. J., Kim H. G., Kim J. S. (2013). The effect of gut microbiota on drug metabolism. *Expert Opinion on Drug Metabolism & Toxicology*.

[44] Stringer A. M., Gibson R. J., Logan R. M., Bowen J. M., Yeoh A. S. J., Keefe D. M. K. (2008). Faecal microflora and *β*-glucuronidase expression are altered in an irinotecan-induced diarrhoea model in rats. *Cancer Biology & Therapy*.

[45] Wang S., Wang M.-L., Li Y., Zhou Y.-B., Wang D.-S. (2012). Clinical study on risk factor associated with gut flora change in patients with rectal cancer during perioperative period. *Zhonghua Wei Chang Wai Ke Za Zhi*.

[46] Takakura A., Kurita A., Asahara T. (2012). Rapid deconjugation of SN-38 glucuronide and adsorption of released free SN-38 by intestinal microorganisms in rat. *Oncology Letters*.

[47] Wallace B. D., Wang H., Lane K. T. (2010). Alleviating cancer drug toxicity by inhibiting a bacterial enzyme. *Science*.

[48] Taha M., Sultan S., Nuzar H. A. (2016). Synthesis and biological evaluation of novel *N*-arylidenequinoline-3-carbohydrazides as potent *β*-glucuronidase inhibitors. *Bioorganic and Medicinal Chemistry*.

[49] Kong R., Liu T., Zhu X. (2014). Old drug new use—amoxapine and its metabolites as potent bacterial *β*-glucuronidase inhibitors for alleviating cancer drug toxicity. *Clinical Cancer Research*.

[50] Ahmad S., Hughes M. A., Yeh L.-A., Scott J. E. (2012). Potential repurposing of known drugs as potent bacterial *β*-glucuronidase inhibitors. *Journal of Biomolecular Screening*.

[51] Narita M., Nagai E., Hagiwara H., Aburada M., Yokoi T., Kamataki T. (1993). Inhibition of *β*-glucuronidase by natural glucuronides of *Kampo*, medicines using glucuronide of SN-38 (7-ethyl-10-hydroxycamptothecin) as a substrate. *Xenobiotica*.

[52] Fujita D., Saito Y., Nakanishi T., Tamai I. (2016). Organic anion transporting polypeptide (OATP)2B1 contributes to gastrointestinal toxicity of anticancer drug SN-38, active metabolite of irinotecan hydrochloride. *Drug Metabolism and Disposition*.

[53] Chen C., Tao C., Liu Z. (2015). A Randomized Clinical Trial of Berberine Hydrochloride in Patients with Diarrhea-Predominant Irritable Bowel Syndrome. *Phytotherapy Research*.

[54] Chen C., Yu Z., Li Y., Fichna J., Storr M. (2014). Effects of berberine in the gastrointestinal tract—a review of actions and therapeutic implications. *American Journal of Chinese Medicine*.

